# Personality disorders do not affect treatment outcomes for chronic HCV infection in Spanish prisoners: the Perseo study

**DOI:** 10.1186/s12879-015-1102-x

**Published:** 2015-08-19

**Authors:** Andrés Marco, José J. Antón, Joan Trujols, Pablo Saíz de la Hoya, José de Juan, Inmaculada Faraco, Joan A Caylà, The Perseo Group

**Affiliations:** Barcelona Men’s Penitentiary Health Services, Barcelona, Spain; CIBER Epidemiology and Public Health (CIBERESP), Barcelona, Spain; Albolote Penitentiary Health Services, Granada, Spain; Department of Psychiatry, Hospital de la Santa Creu i Sant Pau, Sant Pau Biomedical Research Institute (IIB Sant Pau), CIBER Salud Mental (CIBERSAM), Barcelona, Spain; Fontcalent Penitentiary Health Services, Alicante, Spain; Córdoba Penitentiary Health Services, Córdoba, Spain; Sevilla Penitentiary Health Services, Sevilla, Spain; Epidemiology Service, Barcelona Public Health Agency, Plaza Lesseps 1, 08023 Barcelona, Spain

## Abstract

**Background:**

The link between infection with hepatitis C virus (HCV) and personality disorders (PD) has not been investigated in detail. The aim of this study was to compare the effectiveness of HCV treatment in prisoners with and without PD.

**Methods:**

We performed a prospective multicentre study in inmates from 25 Spanish prisons who had been treated with pegylated interferon alfa-2a plus ribavirin in 2011. PD diagnosis was based on the Personality Diagnostic Questionnaire-4+. We calculated adjusted Odds Ratios (AOR) and 95 % confidence intervals (95 % CI) using logistic regression.

**Results:**

The sample included 236 patients (mean age: 40.3 years, 92.8 % male, 79.2 % intravenous drug users, and 26.3 % HIV-coinfected). The prevalence of PD was 72.5 %. 32.2 % of patients discontinued treatment; this percentage was higher in patients with HCV genotypes 1/4 (AOR = 3.55; CI:1.76–7.18) and those without PD (AOR = 2.51; 1.23–5.11). Treatment discontinuation was mainly for penitentiary reasons (40.3 %): release or transfer between prisons.

The rate of sustained viral response (SVR) was 52.1 % by ITT and 76.9 % by observed treatment (OT). SVR was higher among patients with genotype 2 or 3, and those with low baseline HCV-RNA. We did not observe any differences between individuals with and without PD in term of SVR, HCV genotype or HIV infection.

**Conclusions:**

Our results support the safety and clinical effectiveness of the treatment of chronic HCV infection in correctional facilities, both in prisoners with PD and those without. Our data support non-discrimination between patients with and without PD when offering treatment for HCV infection to prison inmates.

**Trial registration:**

Trial registration number (TRN) NCT01900886. Date of registration: July 8, 2013

## Background

There is a strong link between psychiatric disorders and infection with hepatitis C virus (HCV) because patients with psychiatric illness and/or a history of substance abuse are more likely to be infected with HCV. This link is also driven by the fact that, while some psychiatric disorders are due to primary psychiatric illness, they can also result from the HCV infection itself, or be secondary to interferon treatment [1, 2]. Thus, psychiatric pathology is common in individuals infected with HCV, is sometimes severe, and occasionally interferes with treatment for chronic HCV infection. This issue has generated concern in the scientific community: The European Liver Patients Association organised a conference to review the literature and develop expert recommendations for the management of mental health problems in patients infected with HCV [2].

Many injecting drug users (IDU) and/or mentally ill patients end up in prison. Correctional systems have a constitutional obligation to provide adequate health care to inmates, including HCV management. Most prison systems have developed protocol-driven strategies for treating HCV infection in patients, and there are currently no contraindications for the use of pegylated interferon-ribavirin [3]. In Spanish prisons, for example, treatment for chronic HCV infection was introduced in 2002, and has now become part of routine clinical practice. The prevalence of HCV infection among prisoners in Spain is 22.7 %, and 79.8 % of the infected patients are IDU [4]. Many of these HCV-infected inmates suffer from personality disorders (PD) [5, 6], a class of mental disorders characterized as an “enduring pattern of inner experience and behaviour that deviates markedly from the expectation of the individual’s culture, is pervasive and inflexible, is onset in adolescence or early adulthood, is stable over time, and leads to distress or impairment” [7]. Patients with PD are less likely to be treated for chronic HCV infection [8], and, when started, both the adherence and response to treatment are expected to be poorer [9]; however, there are conflicting data on this issue [10]. There is no contraindication for HCV treatment for Spanish prisoners with PD, although there has been little research to date on the link between HCV-infection and PD. The objective of this study was to determine whether PD influences the clinical effectiveness, safety, or rate of discontinuation of treatment with pegylated interferon (Peg-IFN) plus ribavirin in a sample of prisoners with a high prevalence of PD.

## Methods

### Study design and setting

We conducted a prospective, multicentre, open-label, stratified study, with drugs administered under conditions approved for the treatment of chronic HCV infection.

### Study population

All inmates, with or without PD, who were treated with Peg-IFN α2a plus ribavirin at 25 prisons in Spain between 01.01.2011 and 31.12.2011, and who met the following inclusion criteria: a) age ≥18 years; b) expected to stay in prison >18 months; c) undergoing treatment for chronic HCV infection according to routine clinical practice criteria; d) inmate gave written informed consent to participate in the study. The following patients who received treatment were excluded from the study: a) those with significant difficulties in reading, writing and/or understanding or interpreting texts, thus preventing the diagnosis of PD using the Personality Diagnostic Questionnaire-4+ (PDQ-4+); b) those who had been previously treated with interferons, either alone or in combination with ribavirin; and c) cases in which the clinician decided to shorten or lengthen the treatment for chronic HCV infection.

All patients were treated with peg-IFN α2a (180 μg/week) plus daily weight-adjusted or fixed doses of 800 mg ribavirin for 24 or 48 weeks, according to HCV genotype, according to standard recommendations at the time when treatment was initiated. Patients were also treated with opioid maintenance treatment where indicated, and anti-retroviral therapy in those coinfected with HIV. Patients were stratified according to the presence or absence of PD, and the efficacy, safety and rate of discontinuation of treatment with peg-IFN plus ribavirin were analysed in each branch. Effectiveness was evaluated according to sustained viral response (SVR), defined as qualitative HCV RNA <50 IU/ml (Cobas Amplicor HCV Test v2.0, Roche Diagnostic Systems, Barcelona, Spain) 24 weeks after the end of treatment.

### Chart review and HCV parameters

We used patients’ medical histories as a source of information. We collected data on i) socio-demographic and penitentiary variables: age, gender, race, and the year of their first stay in prison; ii) variables related to the use of alcohol and/or drugs: lifetime history of alcohol abuse, lifetime and recent (previous year) intravenous drug use (IDU); and iii) clinical variables: degree of hepatic fibrosis (FIB), HIV infection, HCV RNA level, and HCV genotype.

HCV genotype was determined using a commercial reverse hybridization assay (InnoLipa HCV 2.0, Innogenetics, Ghent, Belgium). HCV RNA was assayed using the Cobas Amplicor HCV Test v2.0 kit (Roche Diagnostic Systems, Barcelona, Spain). Patients were grouped into two categories according to RNA-HCV levels at baseline: high (>400,000 IU/ml) and low (≤400,000 IU/ml). FIB was determined using transition elastography (FibroScan®, Echosens, Paris, France) or liver biopsy. Patients were considered to have advanced FIB if they had grade ≥3, according to liver biopsy or FibroScan® results (≥9.5 kPa).

### A two-step screening and diagnostic instrument for personality disorders

For screening and subsequent diagnosis of PD, we used the Spanish version of the PDQ-4+ [11, 12], which assesses the 10 PDs on the DSM-IV-TR Axis II. The DSM-IV-TR [7] organises PDs into 3 higher-order clusters: Cluster A, the so-called odd/eccentric cluster, is composed of Paranoid, Schizoid and Schizotypal PDs; Cluster B, the so-called dramatic/erratic cluster, encompasses Histrionic, Narcissistic, Borderline and Antisocial PDs; and Cluster C, the so-called anxious/fearful cluster, includes Avoidant, Dependent and Obsessive-Compulsive PDs. The PDQ-4+ consists of a self-report questionnaire (SRQ) and a clinician-administered “Clinical Significance Scale” (CSS). This instrument has demonstrated satisfactory psychometric reliability in the general population [13], in psychiatric outpatients [14], and among prisoners [15–17].

### Procedure

Potential cases of PD were initially detected using the SRQ, and the diagnosis was subsequently confirmed by the interviewer using the CSS. The PDQ-4+ was administered to all inmates who were to receive treatment for chronic HCV infection. The SRQ was filled out during the consultation and was considered valid for assessment when indicated by the scores on both validity scales. All clinicians had previously received training in administering the PDQ-4+ from a psychiatrist and a clinical psychologist.

### Statistical analyses

We performed a descriptive analysis of the socio-demographic and clinical variables, and calculated the mean and standard deviation for continuous variables, and the number and percentage of cases for each response category of categorical variables. The overall prevalence of PD was calculated as the number of patients with chronic HCV infection with some PD according to PDQ-4+, divided by the total number of the total number of eligible patients. The same approach was used to calculate the specific prevalence of each PD.

We assessed clinical effectiveness, safety, and rate of discontinuation of treatment. The results were analysed using intention-to treat (ITT) analysis (we analysed the results for all patients assigned to each treatment group, regardless of whether they completed the treatment and/or follow-up) and by observed treatment (OT) analysis (we analysed the results for patients assigned to each group and who completed the treatment and/or follow-up).

All demographic, epidemiological and clinical variables were analysed separately in individuals with and without PD. Factors with a *p* < 0.2 for association in the bivariate analysis were included as independent variables in the logistic regression analysis. We calculated odds ratios (OR), adjusted odds ratios (AOR) and 95 % confidence intervals (95 % CI); a p-value of ≤0.05 was considered to be statistically significant. All statistical analysis were performed using SAS version 9.1.

### Ethical aspects

This study was approved by the Clinical Research Ethics Committee of Hospital Virgen de las Nieves, Granada, Spain (Code GEI-HCC-2010-01), and was performed with the authorisation of the relevant public administrations responsible for the management of prisons, the Spanish Government’s General Secretariat of Penitentiary Institutions, and the Catalan Government’s Department of Justice.

Eligible inmates were given written information about the content and objectives of the study, and, following a personal interview, were asked to give written informed consent to participate. Before providing consent, all subjects were specifically reminded, as stated on the consent form, that they would receive no monetary compensation nor penitentiary benefits in return for their participation, and that their responses would have any negative effects on their healthcare rights or on their status within the prison.

## Results

**Fig. 1:**
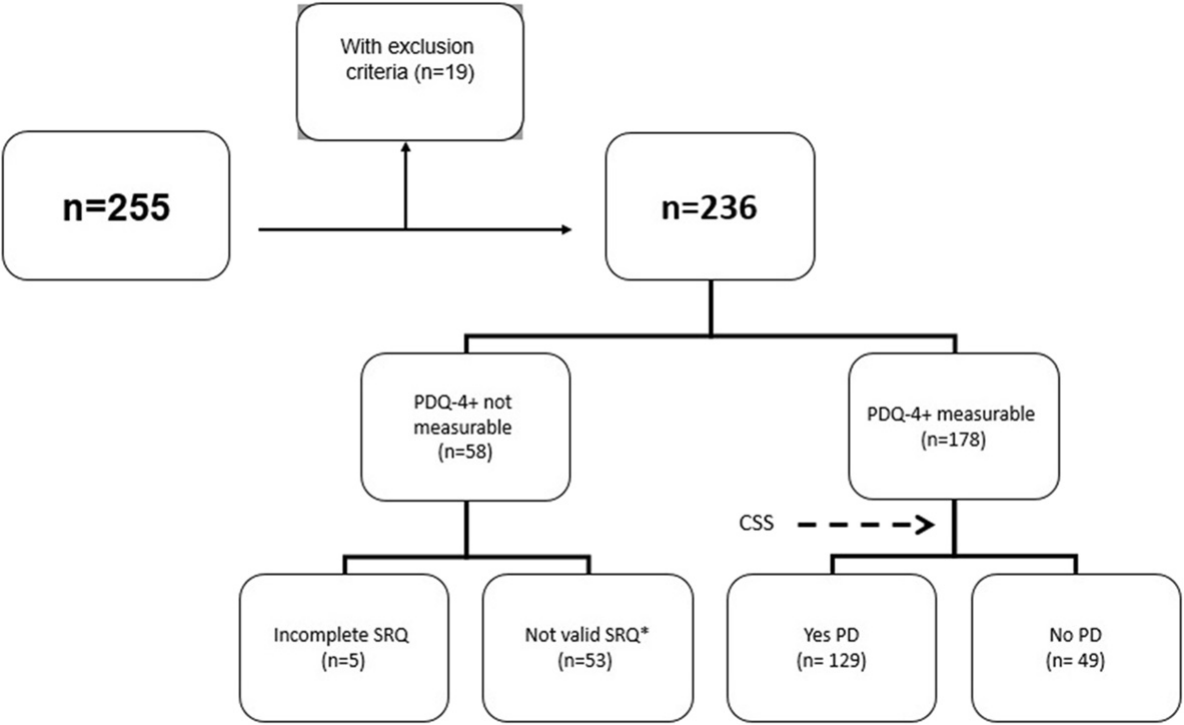
Summary of patient selection process. *Depending on the score obtained on any of the validity scales. PDQ-4+: Personality Diagnostic Questionnaire-4+; PD: personality disorders; CSS: clinical significance scale; SRQ: self-reported questionnaire

Treatment was indicated for 255 prisoners (Fig. 1), 19 of whom (7.4 %) did not meet the inclusion criteria, and were excluded from the analysis. The mean age of the remaining 236 subjects was 40.3 ± 5 years. Of these, 92.8 % were male, 87.7 % were Caucasian, 79.2 % had a history of IDU, and 26.3 % were coinfected with HIV. No patient was coinfected with HBV. Other socio demographic and clinical characteristics of the study population are presented in Table 1.

**Table 1:** Socio-demographic and clinical characteristics, and factors associated with treatment discontinuation in a cohort of 236 prisoners

			Bivariate analysis		Multivariate analysis	
Variable	Overall *n* = 236	Treatment discontinuation *n* = 76 (32.2 %)	OR	p-value	p-value	AOR (95 % CI)
Age group (years)						
20–29	15	7 (46.7)	1			
30–39	94	31 (33.0)	0.56	0.31		
40–49	102	27 (26.5)	0.41	0.12		
≥50	25	11 (44.0)	0.90	0.87		
Sex						
Male	219	69 (31.5)	1			
Female	17	7 (41.2)	1.52	0.41		
HIV infection						
Yes	62	25 (40.3)	1.63	0.11		
No	174	51 (29.3)	1			
Genotype						
1 or 4	141	56 (39.7)	1		0.004	3.55 (1.76–7.18)
2 or 3	95	20 (21.1)	0.40	0.003		1
Genotype and HIV infection						
No HIV and G1 or G4	97	39 (40.2)	0.99	0.99		
No HIV and G2 or G3	77	12 (15.6)	0.27	0.001		
HIV infection	62	25 (40.3)	1	1		
History of alcohol abuse						
Yes	54	16 (29.6)	0.86	0.64		
No	182	60 (33.0)	1			
History of IDU						
Yes	187	61 (32.6)	1.10	0.79		
No	49	15 (30.6)	1			
BVL						
Low	88	28 (31.8)	1			
High	148	48 (32.4)	1.03	0.92		
Personality disorder						
Yes	129	38 (29.5)	0.44	0.01		1
No	49	24 (49.0)	1		0.01	2.51 (1.23–5.11)
Not available or missing	58	14 (24.1)	---			
Cluster A-PD:						
Yes	64	20 (31.2)	0.78	0.45		
No	114	42 (36.8)	1			
Not available or missing	58	14 (24.1)	---			
Cluster B-PD						
Yes	101	29 (28.7)	0.53	0.05		
No	77	33 (42.9)	1			
Not available o Missing	58	14 (24.1)	---			
Cluster C-PD						
Yes	55	18 (32.7)	0.87	0.69		
No	123	44 (35.8)	1			
Not available or missing	58	14 (24.1)	---			
Antisocial PD						
Yes	83	22 (26.5)	0.49	0.03		
No	95	40 (42.1)	1			
Not available or missing	58	14 (24.1)	---			

Cluster A-PD: Paranoid, Schizoid and Schizotypal PDs; Cluster B-PD: Histrionic, Narcissistic, Borderline and Antisocial PDs; Cluster C-PD: Avoidant, Dependent and Obsessive-Compulsive PDs

*OR* odds ratio, *CI* confidence interval, *IDU* injecting drug user, *BVL* baseline viral load, *PD* personality disorder, *AOR* adjusted odds ratio

Five individuals did not fully complete the SRQ (Fig. 1), and a further 53 SRQ responses were not valid (in terms of the scores obtained on the validity scales); thus, the presence or absence of PD was assessed in 178 patients. One hundred and twenty-nine (72.5 %) had PD, 65 (50.3 %) had ≥2 forms of PD, and 47 had (36.4 %) ≥3 forms. The most prevalent types of PD were the antisocial (*n* = 59, 45.7 %), borderline (*n* = 39, 30.2 %) and paranoid (*n* = 37, 28.7 %) types.

FIB was advanced in 75 (31.7 %) cases and grade 4 in 35 cases (14.8 %). Daily ribavirin doses were 1000 mg/day in 49.2 % of cases, 1200–1400 mg/day in 30 %, and 800 mg/day in 20.8 %. In this study, 85.5 % of HIV-infected individuals were receiving antiretroviral treatment (ART), the most frequent combination being efavirenz plus tenofovir disoproxil fumarate plus emtricitabine as a single tablet regimen (28.3 %). In addition, 146 (82 %), 87.6 % of cases with PD and 67.3 % of cases with without PD (*p* = 0.03) were already on psychiatric medication.

Individuals who were co-infected with HIV had: a) significantly higher likelihood of being IDUs; b) a longer history of IDU; c) a longer history of HCV infection; d) a higher frequency of HCV genotypes 1 or 4; e) more advanced FIB; and f) a longer period of time since their first imprisonment. However, we observed no significant difference in the viral RNA of the HCV between individuals HCV monoinfection and those with HIV co-infection.

Over two-thirds (69.1 %, *n* = 163) of individuals treated with peg-IFN plus ribavirin showed adverse effects (AE) due to haematological toxicity (39.2 %) and psychiatric causes (19.2 %). AEs were more common in those co-infected with HIV (75.4 % vs. 58.6 % in monoinfected; *p* = 0.01), although we observed no differences between individuals with PD and those without (13.2 % and 16.7 %, respectively; *p* = 0.58). The treatment dose was modified in 40 (16.9 %) cases. Dose modification was also more frequent in individuals who were coinfected with HIV (27.4 % vs. 13.2 % in monoinfected; *p* = 0.01), but we observed no statistically significant difference between individuals with PD and those without (15.5 % and 20.4 %, respectively; *p* = 0.43).

**Fig. 2:**
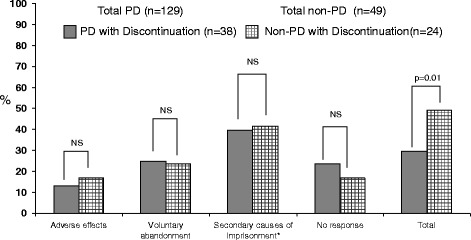
Treatment discontinuation in prisoners with and without personality disorders, stratified by cause of treatment interruption. *Release and/or transfer between prisons. PD: personality disorder; NS: not significant

Seventy-six cases discontinued treatment (32.2 % of the total; 34.8 % of patients who completed the PDQ-4+ evaluation and 24.1 % of those who did not). Treatment was discontinued in 29.3 % (*n* = 51) of HCV monoinfected individuals and 40.3 % (*n* = 25) of coinfected subjects (*p* = 0.11). The rate of discontinuation was higher in patients without PD (49 % vs. 29.5 % in patients with PD; *p* = 0.01), but we observed no difference in the cause of treatment interruption between patients with PD and those without (Fig. 2). Most discontinuations (*n* = 25, 40.3 %) were for penitentiary reasons, i.e., release or transfers between prisons; again, there were no differences in this regard between patients with PD and those without (39.5 % vs. 41.7 %; *p* = 0.86).

In the bivariate analysis, the following variables were associated with discontinuation of treatment: a) absence of antisocial PD; b) absence of B-PD cluster; c) presence of HCV genotype 1 or 4; and d) absence of PD. However, the multi-variant analysis confirmed only the links with genotype 1 or 4, and the absence of PD (Table 1).

While 160 prisoners completed their treatment, SVR could not be verified in 17 because they were released within six months of the end of treatment. Thus, SVR was analysed in 143 subjects. SVR was obtained in 123 cases (52.1 % by ITT, 76.9 % by OT, and 86 % of those who could actually be checked for SVR). The following variables were associated with SVR in the bivariate and multivariate analyses: a) genotype 2 or 3; b) low baseline HCV-RNA (Table 2). We observed no statistically significant differences between cases with and without PD, either globally (51.9 % vs. 46.9 %, *p* = 0.55), or as a function of HCV genotype or the presence or absence of HIV infection.

**Table 2:** Factors associated with sustained viral response in a cohort of 236 prisoners

			Bivariate analysis		Multivariate analysis	
Variable	Overall *n* = 236	SVR *n* = 123 (52.1 %)	OR	p-value	p-value	AOR (95 % CI)
Age group (years)						
20–29	15	7 (46.7)	1			
30–39	94	53 (56.4)	0.68	0.48		
40–49	102	53 (52.0)	0.81	0.70		
≥50	25	10 (40.0)	1.31	0.68		
Sex						
Male	219	114 (52.1)	1			
Female	17	9 (54.9)	0.96	0.94		
HIV infection						
Yes	62	28 (45.2)	1.46	0.20		
No	174	95 (54.6)	1			
Genotype						
1 or 4	141	62 (44.0)	1			1
2 or 3	95	61 (64.2)	0.44	0.002	0.003	0.26–0.76
History of alcohol abuse						
Yes	54	31 (57.4)	0.76	0.38		
No	182	92 (50.5)	1			
History of IDU						
Yes	187	98 (52.4)	0.95	0.86		
No	49	25 (51.0)	1			
BVL						
Low	88	53 (60.2)	1		0.04	1.01–3.04
High	148	70 (47.3)	1.69	0.05		1
Personality disorder						
Yes	129	67 (51.9)	0.82	0.55		
No	49	23 (46.9)	1			
Not available	58	33 (56.9)	---			

*SVR* Sustained viral response, *OR* odds ratio, *CI* confidence interval, *IDU* injecting drug user, *BVL* baseline viral load, *PD* personality disorder, *AOR* adjusted odds ratio

## Discussion

This is the first study, as far as we are aware, that investigates whether PD influences the effectiveness and rate of discontinuation treatment for chronic HCV infection. The study was conducted in a sample of prisoners, many with a history of IDU, and thus with a high the prevalence of PD (72.5 %). This prevalence is higher than the 31 % previously reported in a random samples of patients with HCV [18], but not a great deal higher than the 65 % noted by Fazel and Danesh [5] in their review of studies on HCV-infected prisoners. The prevalence observed in our study is lower than those reported by other studies in prisoners (range: 82–87 %) [19, 20].

The rate of SVR in our study was 52.1 % by ITT and 76.9 % by OT. We did not observe a significant difference in the rate of SVR between subjects with and without PD. The observed rate of SVR was within the range of effectiveness reported in previous studies on prisoners (28–66 %) [21–29], a similar result to those reported in other studies of HCV treatment with peg-IFN α2a plus rivabirin in Spanish prisoners [30, 31]. The observed rate of SVR also is within the range of effectiveness (50–61 %) observed in a meta-analysis aimed at obtaining a pooled estimate of SVR in IDUs [32]; IDUs account for almost 80 % of sample in this study.

Many patients with mental health problems have difficulty in accessing treatment for chronic HCV [33–36], usually for two reasons. First, individuals may fear that peg-IFN may aggravate an existing psychiatric disorder or provoke others, particularly depression and suicide tendencies [37, 38]; however, suicidal ideation occurs in less than 10 % of cases [2, 39] and successful suicide attempts are merely anecdotal [2]. Second, there is a belief that mental health problems during antiviral treatment are risk factors for treatment failure or poorer treatment adherence [40–43]. This last point is assessed when there is a depressive disorder [40–43], although it is more controversial in cases of substance abuse, bipolar disorder or schizophrenia, in light of studies [44–46] demonstrating that chronic HCV infection can be treated safely and effectively in these cases using a multidisciplinary approach [35, 36, 44–48].

To date, there has been limited evidence of the effectiveness of treatment for chronic HCV infection in patients with PD, with only one previous study [10], which did not observe significant differences in tolerability, clinical effectiveness or treatment discontinuation among patients with and without PD; however, this study included only 19 patients with PD. Our study includes a much larger sample as well as a comparison arm, and our results coincide those of the previous study [10] in terms of in tolerability (3.9 % of treatment interruptions due to adverse events, compared to 8.2 % in individuals without PD) and effectiveness (51.9 % of SVR vs. 46.9 % in individuals without PD); however, our we observed higher rates of treatment completion (61.5 % vs. 51 % in cases without PD compared to the previous study [10]. These results support the idea that all patients should receive treatment, regardless of whether or not they have PD.

In general, we observed a higher rate of treatment discontinuation in patients without PD, but no difference in the reasons for discontinuation (adverse effects, voluntary abandonment, or secondary causes of imprisonment) between patients with PD and those without. There is no clear explanation for this observation, although it may be related to the fact that a significantly higher number of participants with PD were already receiving psychiatric treatment before starting treatment for HCV. This could have a protective effect by reducing risk of adverse psychiatric events, and could also result in more frequent contact with health services and consequently lower risk of treatment discontinuation.

In our study, treatment discontinuation was higher in patients with HCV genotype 1 or 4; this result is expected because these cases receive peg-IFN α2 plus ribavirin treatment for a longer period of time, and have higher risk of treatment interruption. In addition, the most common reasons for treatment discontinuation were penitentiary reasons, which accounted for over one-third of discontinuations. In fact, excluding penitentiary reasons, the rate of treatment discontinuation associated with patient compliance, pharmacotherapy or treatment efficacy was very satisfactory: 18.2 % in the entire treated population, and of 17.9 % in patients with PD.

In Spain, the average duration of imprisonment is 19 months [49], such that many prisoners are imprisoned for too short a time to be diagnosed, assessed and treated for chronic HCV infection. In other countries, such as Italy [27] or the U.S. [3], duration of imprisonment is also the main reason for not starting treatment. Our results are consistent with those from other studies demonstrating that the rate of treatment discontinuation can be lowered through better coordination with healthcare services outside prison, which can facilitate treatment completion after release [30, 50]. However, the use of direct-acting antivirals (DAAS), which reduce treatment duration, will also likely reduce the frequency of discontinuation.

We believe that the strengths of this study are i) its prospective nature; ii) the use of a standardised diagnostic procedure, not just a screening approach, to establish the presence or absence of a PD; iii) the large sample size of prisoners and cases with PD (*n* = 236 and *n* = 129, respectively); iv) the multicentre nature of the study (25 prisons) which supports the validity of our results. A limitation is the fact that we have not evaluated the reliability or level of agreement between interviewers in establishing PD status. However, all clinical interviewers, which were allocated in small groups, were trained in administering the PDQ-4+ by the same two experts.

## Conclusion

Our results support the safety and clinical effectiveness of the treatment of chronic HCV infection in correctional facilities, both in prisoners with PD and those without. Thus, our data support non-discrimination between patients with and without PD when offering treatment for HCV infection to prison inmates.

## Abbreviations

PD: Personality disorders
PDQ-4+: Personality diagnostic questionnaire-4+
AOR: Adjusted odds ratio
ITT: Intention to treat
RVR: Rapid viral response
EVR: Early viral response
ETR: End-of-treatment response
SVR: Sustained viral response
OT: Observed treatment
IDU: Intravenous drug users
